# Microvascular decompression in trigeminal neuralgia: predictors of pain relief, complication avoidance, and lessons learned

**DOI:** 10.1007/s00701-021-05028-2

**Published:** 2021-10-21

**Authors:** Johannes Herta, Tobias Schmied, Theresa Bettina Loidl, Wei-te Wang, Wolfgang Marik, Fabian Winter, Matthias Tomschik, Heber Ferraz-Leite, Karl Rössler, Christian Dorfer

**Affiliations:** 1 Department of Neurosurgery, Medical University of Vienna, Währinger Gürtel 18-20, 1090 Vienna, Austria; 2 Division of Neuroradiology and Musculoskeletal Radiology, Medical University of Vienna, Vienna, Austria

**Keywords:** Microvascular decompression, Trigeminal neuralgia, Facial pain, Outcome analysis, Neurovascular contact

## Abstract

**Objective:**

To analyze characteristics associated with long-term pain relief after microvascular decompression (MVD) for trigeminal neuralgia (TGN). Description of associated morbidity and complication avoidance.

**Methods:**

One hundred sixty-five patients with TGN underwent 171 MVD surgeries at the authors’ institution. Patient characteristics and magnetic resonance imaging (MRI) datasets were obtained through the hospital’s archiving system. Patients provided information about pre- and post-operative pain characteristics and neurologic outcome. Favorable outcome was defined as a Barrow Neurological Institute (BNI) pain intensity score of I to III with post-operative improvement of I grade.

**Results:**

Type of TGN pain with purely paroxysmal pain (*p* = 0.0202*) and TGN classification with classical TGN (*p* = 0.0372*) were the only significant predictors for long-term pain relief. Immediate pain relief occurred in 90.6% of patients with a recurrence rate of 39.4% after 3.5 ± 4.6 years. MRI reporting of a neurovascular conflict had a low negative predictive value of 39.6%. Mortality was 0% with major complications observed in 8.2% of patients. Older age was associated with lower complication rates (*p* = 0.0009***). Re-MVD surgeries showed improved long-term pain relief in four out of five cases.

**Conclusions:**

MVD is a safe and effective procedure even in the elderly. It has the unique potential to cure TGN if performed on a regular basis, and if key surgical steps are respected. Early MVD should be offered in case of medical treatment failure and paroxysmal pain symptoms. The presence of a neurovascular conflict on MRI is not mandatory. In case of recurrence, re-MVD is a good treatment option that should be discussed with patients.

**Highlights:**

• Long-term analysis of pain relief after MVD.

• Positive predictors for outcome: classical TGN and purely paroxysmal pain.

• Presence of neurovascular conflict in MRI is not mandatory for MVD surgery.

• Analysis of complications and surgical nuances for avoidance.

• MVD is a safe procedure also in the elderly.

## Introduction

Trigeminal neuralgia (TGN) is a rare disease with an estimated incidence of 12.6 in 100,000 people per year [20]. It is characterized by lancinating paroxysmal pain within the dermatome of the fifth cranial nerve, typically lasting over a short period of seconds to minutes. Mainly unilateral, one or multiple nerve branches can be affected with a predisposition for the mandibular and/or maxillary nerves. Pain attacks may occur spontaneously or be triggered by a number of stimuli, including chewing, speaking, swallowing, or brushing teeth [19]. The resulting pain negatively impacts patients’ quality of life, profoundly impairing daily functioning [35, 41]. The primary therapy of TGN consists of antiepileptic drugs with a strong recommendation for carbamazepine and oxcarbazepine [4]. Response rates are typically high but in cases of poor tolerability or inadequate pain relief, patients should be considered for surgery [38]. Microvascular decompression (MVD) is the first choice in patients with classical and idiopathic TGN who are eligible for posterior fossa surgery [4]. During microsurgery, the prepontine cistern is explored and offending vessels are isolated from the fifth cranial nerve root to prevent further ephaptic connections between demyelinated fibers that might explain the mechanism of triggered pain [10].

The present study is a retrospective single-center analysis of patients with TGN that were operated by MVD. The study tries to clarify (1) the role of neurovascular contact (NVC) in preoperative magnetic resonance imaging (MRI) that has been frequently used as an inclusion criterion for MVD, as arterial compression of the trigeminal nerve root has been associated with better pain freedom rates [17]; (2) the pain recurrence rate after MVD, with a special focus on predictive factors, as pain freedom rates vary between 50 and 89% due to different outcome measures and subgroup analysis utilized across studies as well as center variability [3, 14, 22, 32, 34, 36, 40]; (3) the percentage of associated complications as morbidity and mortality rates of MVD are generally very low but tend to increase with higher age [27, 29]. To prevent complications, surgical nuances are described.

## Methods

### Patient cohort

All patients who received MVD for TGN treatment at the Department of Neurosurgery at the Medical University of Vienna between January 2002 and December 2019 were eligible for enrollment in the present study. Patients underwent MVD if their pain was not sufficiently controlled by medication or if drugs were poorly tolerated as per 2019 guidelines of the European Academy of Neurology [4]. Patients who had previous surgery for TGN, including previous MVD surgeries, were also included in the study. Patients with symptomatic TGN (e.g. multiple sclerosis, schwannoma, aneurysm), incomplete documentation, or missing follow-up examination were excluded from the study.

### Preoperative data collection

Retrospective data collection was obtained from patient admission charts, operative notes, preoperative MRT datasets, discharge letters, and follow-up reports. Preoperative characteristics of patients were documented including age, sex, onset and duration of TGN symptoms, age at surgery, pain distribution, IHS classification of TGN (classical, idiopathic, secondary), category of TGN pain (purely paroxysmal vs. concomitant continuous) [15], type of previous surgical treatment, antiepileptic drug use, and preoperative degree of pain as measured by the Barrow Neurological Institute (BNI) pain intensity score [28]. Patients’ motivation to undergo surgery based on uncontrollable pain, side effects of medication, combination of pain and side effects of medication, and patients’ wish was also assessed.

All patients underwent a preoperative MRI on a 1.5-T or 3-T scanner which included at least one T1-weighted gadolinium sequence, one time-of-flight magnetic resonance angiography sequence, and one high-spatial-resolution T2 sequence (CISS, FIESTA, DRIVE). All images were re-screened to differentiate between idiopathic and classical TGN by a board-certified neuroradiologist according to the severity of NVC in the prepontine cistern as suggested by the American Academy of Neurology in 2016 [9].

### Surgical details of microvascular decompression

Surgical reports were screened for the presence of NVC, the type and name of the compressing vessel, patient positioning during surgery, operation time, and complications during surgery. Surgery was performed depending on the surgeons’ experience and patient requirements in a sitting, prone, supine, or lateral park bench position. Over a retrosigmoid approach, and after opening the dura, the lateral surface of the cerebellum was retracted to open the prepontine or cerebellopontine cistern to release cerebrospinal fluid (CSF). Special attention was drawn to not harm the cerebellum due to extensive retraction or injury to the superior petrosal vein. Following trigeminal nerve exposure, the arachnoid sheet was dissected from the nerve root and the NVC was exposed. The conflicting vessel was carefully transposed and pieces of Teflon™ were placed between the vessel and the trigeminal nerve root. Absence of any offending vessel was ascertained. Surgery was completed after meticulous inspection of the trigeminal root entry zone.

### Postoperative evaluation

Immediate pain relief after surgery as well as at follow-up was the primary outcome parameter and measured by the BNI pain intensity score: (I) no pain, no medication; (II) occasional pain, not requiring medication; (III) some pain, adequately controlled with medication; (IV) some pain, not adequately controlled with medication, and (V) severe pain/no pain relief [28]. Patients were followed in the outpatient clinic and by a written questionnaire. A good outcome was defined as a BNI pain intensity score of I to III if an improvement of I grade compared with the preoperative baseline measure occurred. An unfavorable outcome was defined as a BNI pain intensity score of IV to V, or if no postoperative improvement occurred. Recurrence of pain was defined as recurring pain attack and/or the need of increased medication after the surgery.

Complications were reported and graded into minor and major complications. Minor complications were defined as a BNI facial numbness score ≤ 2, postoperative systemic infections, wound healing disorders not requiring surgical or extensive medical treatment, vertigo, and dry eyes. Major complications were defined as a BNI facial numbness score > 2, hearing impairment, double vision, CSF fistulas, intracerebral/intracerebellar hemorrhage, and extensive brain swelling. Intraoperative bradycardia, which required atropine administration, was considered an expected event during fifth cranial nerve manipulation.

### Statistical analysis

Continuous variables following a normal distribution were assessed by analysis of variance and Student *t*-test. The Mann–Whitney *U*-test was used for non-parametric interval-scaled variables. Chi-square or Fisher exact test was used for categorical data. Kaplan–Meier survival analyses were performed with a time interval from surgery to the date of recurring pain and/or increase of antiepileptic medication. Patients were censored at the last follow-up in the absence of recurrent pain. Several different groups were tested to differentiate significant differences in the probability of freedom of pain: types of NVC, sex, primary surgery versus follow-up surgery, TGN phenotype, affected nerve branches, side of facial pain, and age > 50 years. Survival analysis of pain recurrence was performed by using Mantel-Cox log-rank tests. A *p* value of < 0.05 was considered statistically significant. Statistical verifications were performed using GraphPad Prism (Version 9.1.1).

### Ethical approval

The study protocol was approved by the local ethics committee of the Medical University of Vienna (EK 1092/2020) and is in accordance with the Helsinki Declaration of Human Rights.

## Results

### Patient cohort

Patient characteristics are outlined in Table 1. A total of 165 patients who underwent 171 MVD procedures were included in the study. The median patient age at MVD was 57 years (range: 21–78 years). The median age at TGN onset was 49 years (range: 17–74 years) with a mean time span from TGN onset to MVD as first surgery of 6.7 ± 5 years. Women (53.6%) underwent MVD more frequently. The right trigeminal nerve was more frequently affected with 61.8%. One patient suffered from bilateral TGN but received unilateral surgery. The most affected nerve branches were a combination of the maxillary nerve (V2) and the mandibular nerve (V3) (31%), followed by an isolated involvement of V3 (19.3%) and V2 (18.7%). The predominant reasons for patients to undergo MVD were (1) uncontrollable pain (71.9%), (2) side effects of antiepileptic drugs (3.5%), (3) a combination of (1) and (2) (23.4%), and (4) patient wish even if pain was controlled by medication (1.2%). At least 29.3% of patients had prior unnecessary teeth extractions, but this detail was not documented in 40% of cases.

**Table 1:** Characteristics of trigeminal neuralgia (TGN) patient cohort (n_PAT_ = 165 patients; n_PRO_ = 171 procedures)

Characteristics	Data
Ratio male:female, n_PAT_ (%)	77:88 (46.7%:53.3%)
Age (yr) at TGN onset, median (range)	49 (17–74)
Duration of symptoms (years), mean (SD)	7.6 ± 5.9
Reason of patient to undergo MVD, n_PRO_ (%)	
1 Uncontrollable pain	123 (71.9%)
2 Side effects of medication	6 (3.5%)
3 Combination of 1 and 2	40 (23.4%)
4 Patients wish	2 (1.2%)
Age (yr) at MVD surgery, median (range)	57 (21–78)
Previous neurodestructive surgery, *n* (%)	18 (10.5%)
Previous MVD surgery, n (%)	6 (3.5%)
IHS classification of trigeminal pain - MRI, n_PRO_ (%)	
Classical	67 (39.2%)
Idiopathic	102 (59.6%)
Unknown	2 (1.2%)
Pain category, n_PRO_	
Purely paroxysmal pain, *n* (%)	94 (55%)
Concomitant continuous pain 2, *n* (%)	38 (22.2%)
Not specified	39 (22.8%)
IHS classification of trigeminal pain - intraoperative finding, n_PRO_ (%)	
Classical	129 (75.4%)
Idiopathic	41 (24%)
Unknown	1 (0.6%)
Vessel causing neurovascular conflict at first surgery, n_PAT_ (%)	
Artery	82 (49.7%)
Vein	22 (13.3%)
Artery and vein	55 (33.3%)
No neurovascular conflict	5 (3%)
Unknown	1 (0.6%)
Most frequent arterial compressions, *n* = 135(%)	
SCA	93 (68.9%)
AICA	12 (8.9%)
AICA and SCA	11 (8.2%)
BA	1 (0.7%)
Unspecified	18 (13.3%)
Affected nerve branch, n_PRO_ (%)	
V1 only	1 (0.6%)
V2 only	32 (18.7%)
V3 only	33 (19.3%)
V1 and V2	21 (12.3%)
V1 and V3	2 (1.2%)
V2 and V3	53 (31%)
V1 and V2 and V3	29 (17%)
Affected side, n_PAT_ (%)	
Left	62 (37.6%)
Right	102 (61.8%)
Bilateral	1 (0.6%)
Preoperative BNI score, n_PRO_	
III	5 (2.9%)
IV	24 (14%)
V	142 (83%)
Preoperative facial numbness n (%)	27 (15.8%)

*TGN* trigeminal neuralgia; *MVD* microvascular decompression; *n*_*PAT*_ number of patients; *n*_*PRO*_ number of procedures_;_ *IHS* International Headache Society; *BNI* Barrow Neurological Institute; *V1* ophthalmic nerve; *V2* maxillary nerve; *V3* mandibular nerve; *SCA* superior cerebellar artery; *AICA* anterior inferior cerebellar artery; *BA* basilar artery

### Classification of trigeminal neuralgia

The type of pain was paroxysmal in 94/171 (55%) patients and 38/171 (22.2%) patients experienced concomitant continuous pain. Classification could not be determined in 29/171 (22.8%) patients.

The preoperative MRI scans revealed classical TGN in 67/171 (39.2%) patients and idiopathic TGN in 102/171 (59.7%) patients. Retrospective MRI scans were unavailable in two patients. Two patients suffered from multiple sclerosis but were classified as classical TGN as both MRI scans showed a NVC at the affected side in the absence of multiple sclerosis plaques in the trigeminal nuclei. TGN was classified again by the intraoperative judgment of the treating neurosurgeon. TGN was classified as classical in 129/171 (75.4%) patients and as idiopathic in 41/171 (24%) patients. One patient was not classified by the treating neurosurgeon. Taking these numbers into account, MRI had a detection sensitivity for a NVC of 52% and a specificity of 97.6%, giving a low negative predictive value of 39.6%. Intraoperatively, the NVC was caused by an artery in the majority of cases (49.7%), namely, the superior cerebellar artery (68.9%) followed by a combination of arteries and veins (33.3%) and veins only (13.3%).

### Surgical procedure

A total of 149/171 (87.13%) MVD surgeries were primary surgeries for TGN. The numbers of previous procedures for TGN in the remaining 20 patients (22 MVDs [12.9%]) were the following: 2nd surgery *n* = 12; 3rd surgery *n* = 4; 4th surgery *n* = 2; 5th surgery *n* = 1, 6th surgery *n* = 1, 7th surgery *n* = 2. Twelve patients had one or multiple prior percutaneous ablative procedures at the trigeminal ganglion, two patients had prior gamma knife radiosurgery (GKN) of the trigeminal nerve, three patients had a second MVD, and three patients had a combination of multiple MVDs and ablative procedures.

**Fig. 1:**
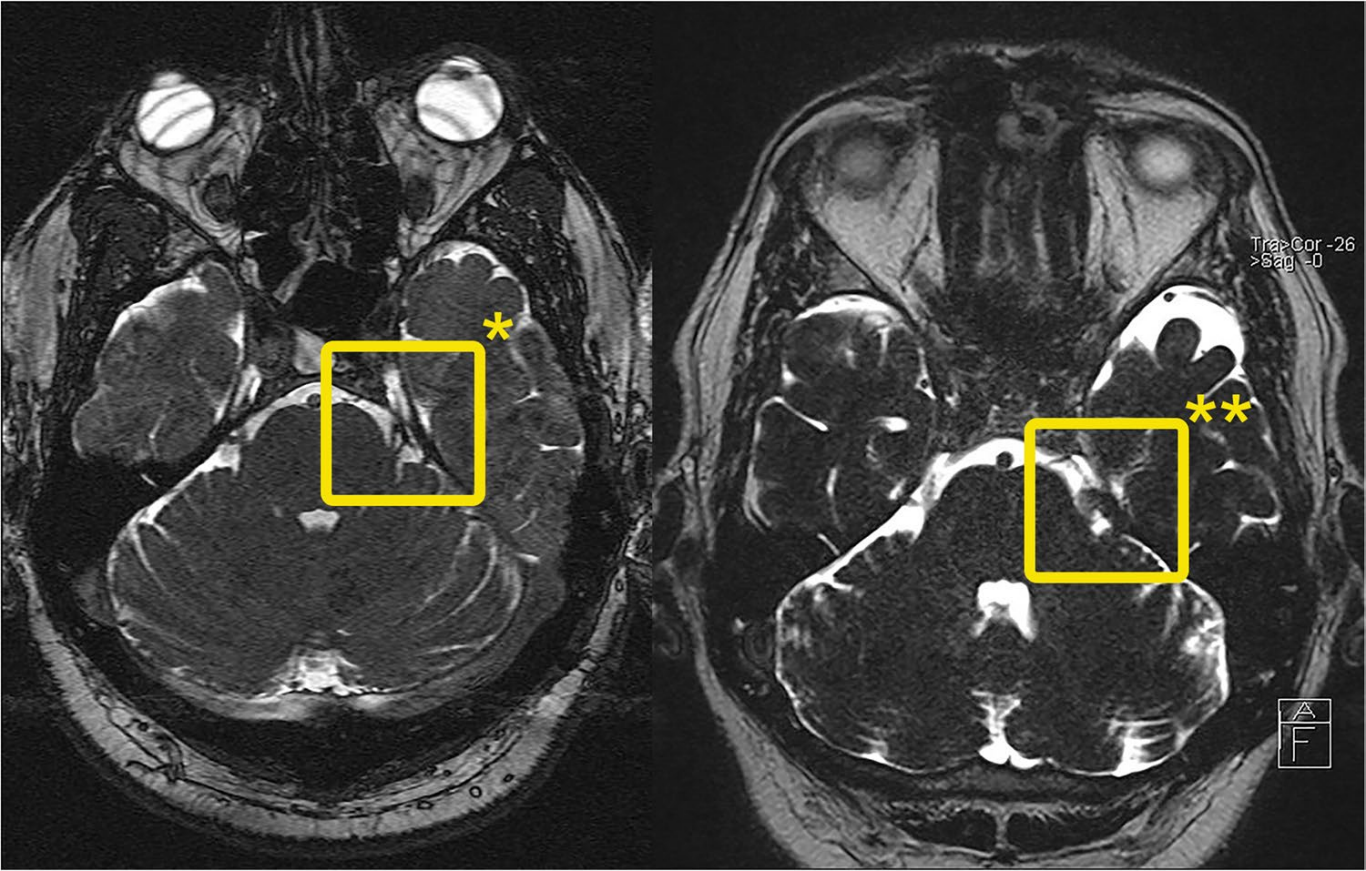
Male patient in his 5th decade of life with classical TGN in the 2nd and 3rd divisions of the left TGN. Following a unsuccessful first MVD at another institution, the patient underwent four subsequent radiofrequency thermocoagulations of the trigeminal ganglion at our clinic resulting in short periods of pain improvement of approximately 1 year and pain reduction with every intervention. Due to these short periods, classic symptoms and a still existing NVC in MRI (*); a second MVD was performed which revealed insufficient decompression and resulted in a BNI pain intensity score of I. Unfortunately, pain recurrence occurred after 2 years in the first division of the left TGN. MRI (**) showed the TGN root surrounded by Teflon™ and the SCA with a further close relationship. After a long consultation, the patient requested another exploration. During the surgical procedure, the nerve-Teflon™ convolute was left in place, the adjacent SCA was sharply dissected away and isolated again, which resulted in a current BNI pain intensity score of I

A total of 5/6 patients who underwent two consecutive MVD surgeries had a favorable outcome at long-term follow-up. The patient with an unfavorable outcome experienced pain recurrence 534 days after surgery. One patient underwent a total of three MVD surgeries (Fig. 1). In cases of re-MVD, the following abnormalities were found: ongoing NVC (*n* = 4), scaring (*n* = 1), and nerve distortion by Teflon™ (*n* = 1).

The mean time for the MVD procedure was 178.7 ± 47.1 min. Patients were hospitalized for 7.3 ± 2.2 days. Patient positioning was in a lateral (82.5%), prone (12.3%), sitting (3.5%), or supine (1.7%) position as determined by the neurosurgeon’s preference. One surgery was terminated early prior to neurovascular decompression due to incorrect patient positioning in the lateral position, which resulted in insufficient venous drainage and brain swelling. Consequently, this patient was excluded from BNI pain intensity score outcome analysis.

Surgeries were performed by fifteen different surgeons over time (Table 2). In total, 50% of surgeries were performed by a single surgeon and 83% of surgeries were executed by surgeons with a high level of experience in cerebellopontine angle surgery. There was no negative correlation between the caseload of MVD and major complications (*r* = 0.81).

**Table 2:** Influence of surgeon’s experience on MVD results

Surgeon ID	Level of experience in CPA surgery	Caseload *n* (%) *n* = 171	Major complications *n* (%) *n* = 14	Pain recurrence *n* (%) *n* = 68
S1	High	85 (49.7)	6 (42.9)	36 (52.9)
S2	Moderate	20 (11.7)	3 (21.4)	6 (8.8)
S3	High	14 (8.2)	1 (7.1)	4 (5.9)
S4	High	12 (7)	2 (14.3)	5 (7.4)
S5	Moderate	12 (7)	0 (0)	3 (4.4)
S6	High	8 (4.7)	0 (0)	3 (4.4)
S7	Low	5 (2.9)	0 (0)	3 (4.4)
S8	Low	4 (2.3)	2 (14.3)	2 (2.9)
S9	Moderate	3 (2)	0 (0)	3 (4.4)
S10	High	2 (1.2)	0 (0)	1 (1.5)
S11	Low	2 (1.2)	0 (0)	0 (0)
S12	High	1 (0.6)	0 (0)	0 (0)
S13	High	1 (0.6)	0 (0)	1 (1.5)
S14	Low	1 (0.6)	0 (0)	0 (0)
S15	Moderate	1 (0.6)	0 (0)	1 (1.5)

*CPA* cerebellopontine angle, *MVD* microvascular decompression

### Postoperative and long-term outcome after MVD

**Fig. 2:**
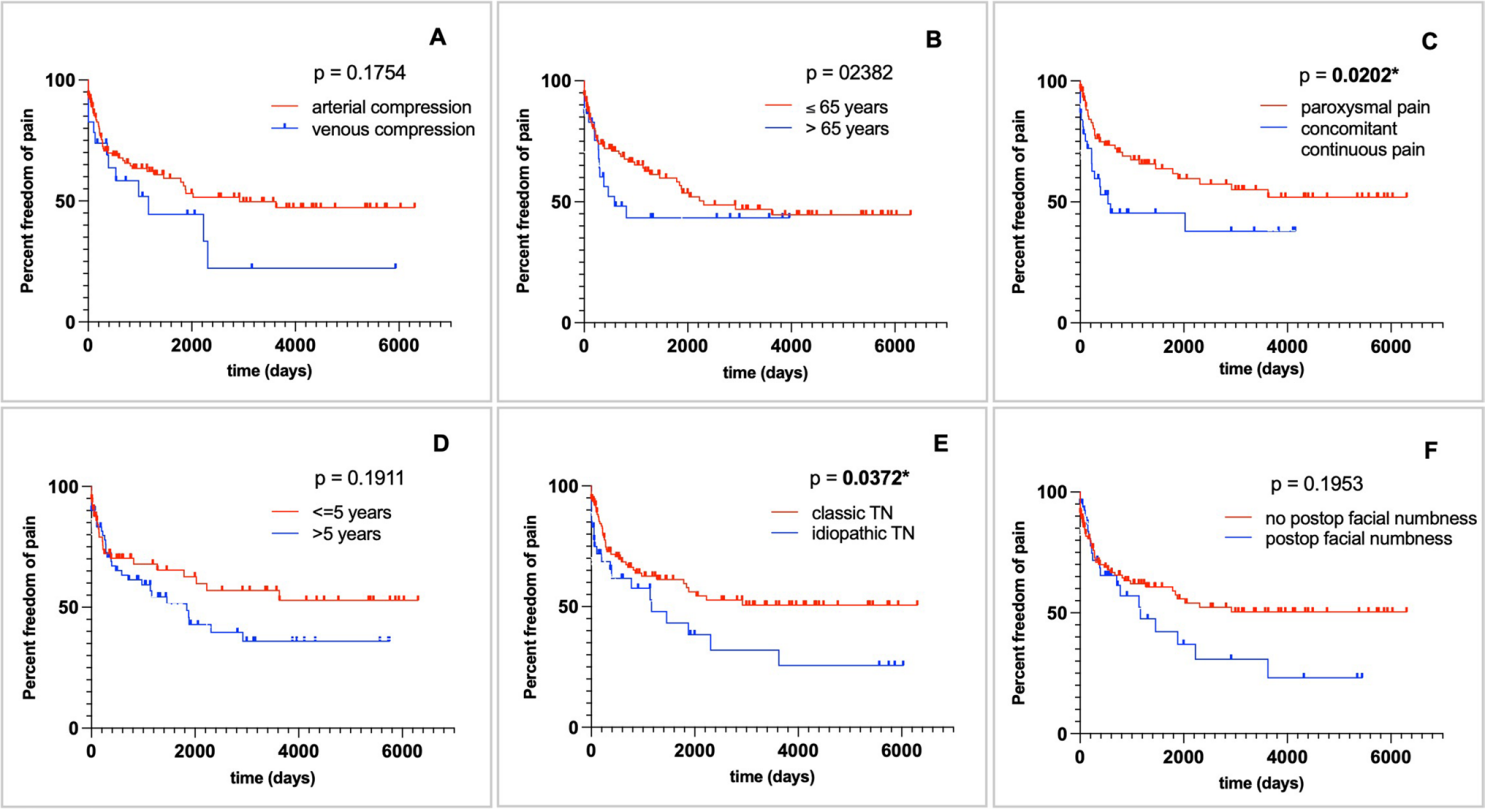
Kaplan–Meier survival curves show the probability of pain freedom over time based on **A** the type of vessel causing the neurovascular contact, **B** the patient age at surgery, **C** the type of trigeminal pain, **D** the duration of TGN symptoms till microvascular decompression surgery, **E** the International Headache Society classification of trigeminal neuralgia, and **F** the occurrence of postoperative facial numbness. Patients were censored at the date of pain recurrence or at the date of last follow-up if pain relief occurred

Pain outcome after MVD was analyzed for 170 surgeries with a mean follow-up of 3.5 ± 4.6 years. Recurrence after MVD appeared in 67/170 (39.4%) cases with a median time from MVD to recurrence of 127 days (range 0–5932 days). A total of 35/66 (53%) patients who suffered from recurrent pain after MVD underwent a subsequent ablative procedure (25 radiofrequency-thermocoagulations, 28 percutaneous balloon compressions, 5 GKN) at our department. Kaplan–Meier analysis of pain recurrence over time is shown in Fig. 2. Significant differences in pain recurrence were found between classical and idiopathic TGN (*p* = 0.0372*) when based on the surgeons intraoperative classification (*p* = 0.0202*), but not when based on preoperative MRI (*p* = 0.1002). The type of vessel causing the NVC (*p* = 0.1754), patient age at surgery (*p* = 0.2382), duration of TGN symptoms until MVD procedure (*p* = 0.1911), occurrence of postoperative facial numbness (*p* = 0.1953), sex (*p* = 0.7981), MVD as primary or subsequent surgery (*p* = 0.7500), side of facial pain (*p* = 0.3654), and involved nerve branches (*p* = 0.3795) were statistically not significant.

**Fig. 3:**
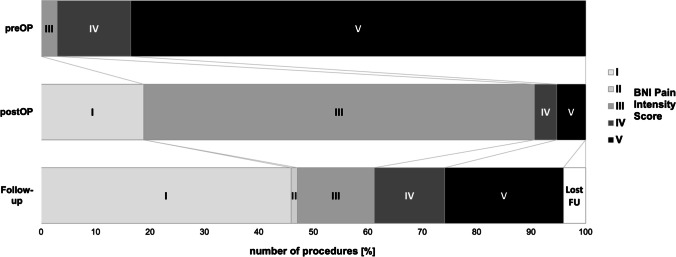
Changes in BNI pain intensity score before (preOP), immediately after (postOP), and at latest follow-up (mean follow-up at 3.5 ± 4.6 years) after microvascular decompression surgery (*n* = 170)

Similar results were found when patients were dichotomized into a favorable and an unfavorable outcome group at long-term follow-up (Table 3). Of the 170 patients at discharge, seven patients got lost during the follow-up, leaving 163 patients for long-term analysis. At discharge, 90.6% of patients were categorized into the favorable outcome group with a BNI pain intensity score of I, II, and III. However, only 63.8% of patients had a favorable outcome at long-term follow-up, which was significantly associated with a shorter duration (6.4 ± 5.3 vs. 7.4 ± 4.4 years) of TGN symptoms until MVD procedure (*p* = 0.0316*). Figure 3 shows the changes in the BNI pain intensity score over time.

**Table 3:** Potential factors associated with long-term pain outcome after microvascular decompression surgery for trigeminal neuralgia (TGN)

Characteristics		Favorable outcome (*n* = 103)	Unfavorable outcome (*n* = 60)	*p* value	n
Age (yr)		56.7 ± 11.7	54.9 ± 9.9	0.3158	163
Sex	Male	49	29	0.9253	163
	Female	54	31		
Duration of symptoms (yr)		6.4 ± 5.3	7.4 ± 4.4	0.0316*	138
Age at disease onset (yr)		49.1 ± 11.1	47.4 ± 9.5	0.3314	161
Pain category	Paroxysmal pain	61	29	0.0228*	125
	Concomitant continuous pain	16	19		
Previous surgery for TGN	No	89	51	0.8034	163
	Yes	14	9		
Nerve branch involvement	Single	37	24	0.6039	163
	Multiple	66	36		
Side of TGN	Right	63	34	0.5226	163
	Left	39	26		
	Bilateral	1	0		
Compression	Artery + Mixed	89	46	0.3342	163
	Vein	11	12		
	No compression	3	2		
BNI facial numbness score	I	84	42	0.1864	164
	II	12	10		
	III	7	8		
	IV	0	1		

*BNI* Barrow Neurological Institute; *TGN* trigeminal neuralgia

The number of MVDs performed by a single surgeon did not correlate with the outcome nor did we see any significant changes over time.

### Complications

The mortality rate as a result of MVD in a total of 171 procedures was 0% with an overall morbidity of 32.8% (*n* = 56). Minor complications occurred in 42/171 (24.6%) procedures compared with 14/171 (8.2%) procedures where major complications occurred. A detailed overview is presented in Table 4. Patient positioning (*p* = 0.6874) and previous surgeries for TGN at the same side (*p* = 0.7230) did not affect the complication rate. Elderly patients were less likely to have complications (*p* = 0.0009***) but were equally affected if only major complications were taken into account (*p* = 0.4607). Patients with major complications (Table 5) versus patients with minor complications were significantly longer hospitalized (*p* = 0.0001***) with a mean duration of 13.6 ± 10.2 versus 7.4 ± 2 days. Intraoperative bradycardia requiring atropine intervention during surgical manipulation of the trigeminal nerve root occurred in 19/171 (11.1%) procedures.

**Table 4:** Number of microvascular decompression surgeries (*n* = 171) accompanied by complications

Complications and side effects	*n* (%)
Mortality	0 (0%)
Minor complications	42 (24.6%)
BNI facial numbness score II	26 (15.2%)
Tinnitus	2 (1.2%)
Vertigo	2 (1.2%)
Intermittent hearing deficit	3 (1.8%)
Wound healing problem	1 (0.6%)
Major complications	14 (8.2%)
CSF fistula	4 (2.3%)
Hygroma	1 (0.6%)
Cerebellar hemorrhage	1 (0.6%)
Cerebellar infarction	2 (1.2%)
Facial palsy	2 (1.2%)
Permanent ipsilateral hearing loss	2 (1.2%)
Trochlear palsy	2 (1.2%)
BNI facial numbness score III	15 (8.8%)
BNI facial numbness score IV	1 (0.6%)
Expected side effects	
Intraoperative bradycardia treated with atropine	*19 (11.1%)*

*MVD* microvascular decompression; *CSF* cerebrospinal fluid

**Table 5:** Analysis of major complications (*n* = 14) in MVD surgery

Patient ID	Age at MVD	ASA Score	Sufficient surgical experience	Patient positioning	Prior ipsilateral posterior fossa surgery	Description of complication	Length of hospital stay	Transfer to another clinic	Long-term Pain outcome
TN00156	64	2	Yes	Lateral	No	Displacement of the trochlear nerve together with the SCA during decompression resulted in a 1-year-long trochlear nerve palsy with double vision	8	No	Good; BNI III
TN00183	67	2	Yes	Lateral	Yes	CSF fistula after second posterior fossa surgery without bone replacement and atrophic neck muscles makes revision surgery necessary	16	No	Bad; BNI IV
TN00224	68	2	Yes	Lateral	no	Intraoperative coagulation of multiple draining veins causes venous cerebellar congestive bleeding that does not require any surgical intervention. Increased hospitalization due to postoperative decompensation of a pre-existing bilateral vocal cord paralysis after resection of a squamous cell carcinoma, neck dissection and radiation which necessitates a tracheostomy	45	ENT	Good; BNI I
TN00272	59	2	Yes	Lateral	No	Development of a right hemispheric hygroma with pronounced mid-line shift 3 weeks after discharge led to a soporous condition and an emergency operation with emptying via a burr hole	8	No	Good; BNI I
TN00273	62	2	No	Prone	No	Intraoperatively confusing situation with unintentional opening of the sigmoid sinus. The vestibulocochlear nerve was mistaken for the trigeminal nerve. MVD lead to a left-sided AICA infarction with accompanying facial palsy (House-Brackmann Grade III, ipsilateral hearing loss, dizziness and a tendency to fall to the right	10	Neurorehabilitation	Good; BNI I
TN00283	59	2	Yes	Lateral	No	Insufficient patient positioning causes massive venous congestion and brain swelling that cannot be controlled by intraoperative cerebellopontine CSF drainage and repositioning of the head. Abort of the operation. No neurologic deficits	6	No	Bad; BNI III
TN00290	51	2	Yes	Lateral	Yes	CSF fistula that heals after a lumbar drain has been placed for five days	14	No	Bad; BNI V
TN00322	59	2	Yes	Supine	No	Opening of mastoid cells leads to rhinoliquorrhea and consecutive revision surgery despite covering with muscle and fibrin glue	18	No	Good; BNI I
TN00333	57	1	Yes	Prone	No	No NVC. Combing of the trigeminal nerve root results in a BNI facial numbness score of IV	8	No	Bad; BNI V
TN00340	51	1	Yes	Lateral	No	Postoperative temporary trochlear palsy which makes it necessary to wear prism glasses for a short time. The nerve was not seen intraoperatively. No abnormal findings in postoperative imaging	8	No	Good; BNI I
TN00344	42	1	Yes	Lateral	No	Postoperative ipsilateral hearing loss. No abnormal intraoperative findings. No abnormal findings in postoperative imaging	8	No	Good; BNI I
TN00366	38	1	Yes	Lateral	Yes	Successful treatment of CSF fistula after a lumbar drain had been placed for six days	17	No	Good; BNI I
TN00399	40	1	No	Lateral	Yes	Second MVD makes it necessary to loosen scarring with otherwise normal operative course. Postoperative facial paralysis (House-Brackmann Grade II) without abnormal findings in the postoperative CT scan. Clinical deterioration on the fifth postoperative day with pronounced ataxia and a wide-legged gait. The MRI examination showed a small, demarcated, subacute infarct area pontocerebellar without bleeding	8	Neurorehabilitation	Good; BNI I
TN00406	34	2	Yes	Lateral	No	Opening of mastoid cells leads despite covering with muscle, fibrin glue and TachoSil® to rhinoliquorrhea at postoperative day three. Lumbar drain was successfully placed for 7 days	11	No	Bad; BNI IV

*AICA* anterior inferior cerebellar artery; *ASA score* American Society of Anesthesiologists score; *BNI* Barrow Neurological Institute; *CSF* cerebrospinal fluid; *MVD* microvascular decompression; *SCA* superior cerebellar artery

The number of MVDs performed by a single surgeon did not correlate with the complication rate.

## Discussion

### Classification of trigeminal neuralgia

The calculated MRI sensitivity and specificity in correctly distinguishing idiopathic from classical TGN were only 52% and 97.6%, respectively, when comparing preoperative MRI findings with intraoperative findings. This means that of 101 supposedly idiopathic TGN patients classified by MRI, a total of 61 (60.4%) patients showed a NVC intraoperatively. This low MRI sensitivity is partially attributed to the etiological classification of TGN as proposed by the American Academy of Neurology in 2016 [9]. Based on their definition, classical TGN is “a neurovascular compression with morphological changes of the trigeminal root” and idiopathic TGN is a TGN with “no neurovascular contact (NVC) or NVC without morphological changes of the trigeminal root” [15]. More gradual classification schemes, such as the MRI grading system proposed by Leal et al., may result in greater accuracy [21]. However, as shown by Brînzeu et al., even with these more sophisticated evaluation schemes, MRI underestimated a NVC in about 45% of cases. Furthermore, 90% of these underestimations were intraoperatively caused by root displacements, distortions, or indentations [6]. As it is generally accepted that patients with classical TGN benefit more from MVD than patients with idiopathic TGN, relying on the MRI in counseling patients with regard to the potential MVD outcome may be misleading [16]. In our series, only the intraoperative but not the MRI classification was predictive of pain relief. Hence, our results emphasize that the presence of a NVC on MRI is not mandatory to suggest MVD to the patient.

Multiple sclerosis is one of the most common causes of secondary TGN. Due to its uncertain pathophysiological mechanism, treatment by MVD is still under debate. In our cohort, two patients with multiple sclerosis were treated by means of MVD procedure. Both patients revealed a NVC and showed immediate pain relief after MVD, while only one patient remained pain-free at long-term follow-up. To date, only few studies investigated patients with multiple sclerosis who were offered MVD. However, all concluded that, even in the presence of multiple sclerosis, MVD is an effective treatment option that should be considered [2, 7, 13, 31]. Based on discrepancies in long-term patient outcome, Paulo et al. suggested to carefully select patients who do not reveal a demyelinating plaque near the trigeminal nucleus [26].

Furthermore, the International Headache Society differentiates two phenotypes of TGN: (1) purely paroxysmal TGN and (2) TGN with concomitant continuous pain [9]. In our analysis, patients with purely paroxysmal pain had significantly better long-term pain relief (*p* = 0.0202*), which is consistent with previous studies [17].

### Surgical procedure

MVD should be the surgical treatment of choice in medical refractory idiopathic and classical TGN. MVD was not performed as the first surgical intervention in 14 patients. The reasons for this were (1) rejection of MVD by the patient, (2) incorrect counseling of the patient by physicians who were inexperienced in the management of TGN, and (3) a missing NVC in cranial MRI and consecutive refusal to perform MVD by the treating neurosurgeon. All reasons indicate that patients who require surgical treatment for TGN should be counseled and treated in specialized centers that offer multiple therapy modalities. Surgeons should clarify that MVD is the sole causative treatment option that has proven to be an effective, low-risk surgery that can be performed even in the absence of a NVC in MRI.

Re-exploration in recurrent TGN in our cohort has shown a success rate of 66% (favorable outcome) and was not associated with a higher complication rate. Similar results have been found by Hussain et al., reporting results in 32 patients who underwent re-exploration of MVD or intraoperative neurolysis (*n* = 11) after a successful primary surgery. They found that after second surgery, 87% and 50% of patients had an improved BNI pain intensity score of I or II, respectively, with no significant complications. In concordance with our data, the major cause for TGN was ongoing compression from an artery or vein followed by nerve distortion and displacement secondary to scarring or adhesions [18]. The indication for re-exploration should be chosen carefully and on an individual basis by the surgeon who performed the first MVD. We follow the concept that in MVD one has one chance only to do it right and, therefore, should be absolutely confident about the completeness of the intraoperative result before closure. Therefore, re-exploration should always be critically questioned and appropriate patient counseling is pivotal in these situations.

### Immediate and long-term outcome after MVD

A total of 90.6% of patients had a favorable outcome with a BNI pain intensity score of I to III at discharge (Fig. 3) compared with only 63.8% of patients after a mean follow-up time of 3.5 ± 4.6 years. Previous studies reported similar immediate pain relief in 84% to 98% of patients, but found a lower recurrence rate in many of these patients [3, 8, 30, 32, 34, 36, 37, 39, 40, 42]. This may reflect the fact that, due to a lack of more restrictive policies at our institution, patients were treated by 15 different surgeons with varying level of experience. A recent meta-analysis of 3897 patients revealed an overall pain freedom rate of 75.8% (BNI pain intensity score I) at a follow-up time of 1.7 ± 1.3 years [17]. Predictors of pain freedom were identified as disease duration of less or equal 5 years, arterial compression, superior cerebellar artery involvement, and paroxysmal pain prior to surgery [17]. In our survival analysis, the duration of symptoms of more than 5 years showed a trend towards a rate of pain recurrence, which was found to be significant between the favorable and unfavorable outcome groups (Fig. 2D). Central sensitization of neurons in the spinal trigeminal nucleus due to the over-excitability of primary afferents may explain the poorer response rate after MVD in the case of persistent symptoms [12, 24]. Early surgical intervention is therefore considered advantageous, which is further supported by our successful results in patients who underwent surgery despite being refractory to conservative therapy.

Arterial compression is generally accepted to play a pivotal role in the pathophysiology of this disease and has been shown to be the strongest predictor for a good outcome (odds ratio = 3.35) [17]. Even though a trend was found in our series, arterial compression did not reach statistical significance in order to predict a favorable outcome. Nonetheless, the pain freedom rate was significantly higher in patients with classical TGN than in patients with idiopathic TGN.

Direct comparisons between individual studies regarding patients’ outcome after MVD are limited by inconsistent definitions for a successful surgery. The definition of pain recurrence was interpreted very strictly in our study. Every postoperative pain attack or added pain medication was rated as pain recurrence, even in the case of longer pain-free periods. In a prospective trial Sindou et al. published their long-term results of 362 patients treated by MVD. Their total cure rate was estimated to be 73.4% at 15 years [34]. Interestingly, the authors described high fluctuations of pain symptoms in the first year after surgery, with 13% of patients considered a surgical failure and slowly achieving pain relief without medication.

### Complications and lessons learned

With mortality rates of less than 0.3% and low morbidity rates of approximately 7–9% (excluding herpes simplex and facial hypesthesia), MVD is not only an effective but also a relatively safe procedure [4]. Our mortality and morbidity rates correspond to those of international studies [3, 7, 25]. A meta-analysis by Sekula et al., which compared the complications between young and old patients (*n* = 1334) from eight studies, could not demonstrate a significantly higher complication rate in the elderly [33]. In contrast, Rughani et al. observed a higher in hospital mortality and a higher complication rate for CSF fistulas and wound healing problems in 1020 patients over the age of 65 years. They concluded that higher age could be used as a surrogate parameter for higher complication rates [29]. Interestingly, in our study, older patients had significant less complications (*p* = 0.0009***). This may be due to increased cerebellar atrophy in older age, which makes it easier for the surgeon to get an overview of the cerebellopontine junction. Another interesting result was with regard to the occurrence of postoperative facial hypesthesia. In contrast to destructive, percutaneous interventions, in which postoperative facial numbness may be a positive predictor for long-term postoperative pain freedom, the opposite may be the case in MVD surgery as shown in Fig. 2F.

Several technical implementations have been discussed by Bond et al. to avoid complications (Table 5) such as ipsilateral hearing loss in MVD [5]. The authors promote the use of brainstem auditory evoked potentials (BAEP), which have been shown to decrease ipsilateral hearing loss from 1.98% before to 0.8% after its implementation in a large cohort of patients (*n* = 4400) [23]. In our cohort, we did not use BAEP for MVD. In both (1.2%) cases of permanent hearing loss, patients may have had a major benefit from BAEP. In the first case, postoperative hearing loss could not be explained by the operative course nor by postoperative imaging. In these cases, cerebellar retraction, vascular injury, and injury to the vestibulocochlear nerve complex are the most common conditions that are preventable by BAEP. In the second case, due to an unclear operative situs, the vestibulocochlear nerve was mistaken for the TGN and decompressed by a neurosurgical team without a lot of experience in cerebellopontine angle surgery. Consequently, the patient developed an infarction in the territory of the anterior inferior cerebellar artery (AICA). This error would have been noticed at an early stage if intraoperative neuromonitoring had been utilized. However, we propose that a solid knowledge of the anatomy is more crucial than the use of IOM in order to ensure a safe surgical procedure.

As in all neurosurgical procedures, patient positioning is absolutely vital. At our institution, the supine, prone, sitting, and lateral park bench positions were employed, depending on the neurosurgeon’s preference. There is certainly no exclusively optimal solution for MVD and the best option depends on various factors. These factors include, above all, the individual patient physiognomie, the surgeon’s experience, and the side of the operation. Sufficient venous drainage, which was not the case in three patients (leading to difficulties during surgery with one aborted surgery and the above mentioned AICA territory infarction), is even more crucial than the type of positioning. A lumbar drain is not mandatory to guarantee a relaxed cerebellum. Based on our experience, we suggest that the sitting position provides the best option in pyknic and overweight patients with a short neck because (1) it avoids venous congestions leading to cerebellar swelling and (2) it provides the best overview of the cerebellopontine cisterns and ameliorates surgeons comfort. A prerequisite is the surgeon’s and institutional experience with the sitting position. For the prone position as well as the lateral decubital position, it is mandatory to elevate the upper body as much as possible to avoid venous congestion. The prone position has the advantage that the patient’s shoulder comes out of the surgeon’s view, which is especially important in case of left-sided MVD and a right-handed surgeon. Taking these critical factors into account, cerebellar retraction should not be necessary in any patient.

Optimization of the operative corridor starts with an optimal skin incision, which is dictated by the patient’s physiognomy and positioning. The aim should be to keep the incision as small as possible while obtaining adequate exposure for the craniotomy, a wide view of the anatomical structures, and freedom for manipulation of the cerebellopontine cistern. A craniotomy perfectly placed at the edge of the transverse and sigmoidal sinus angle does not need to be larger than 2 cm in diameter. It should clearly visualize the venous angle and allow a supracerebellar approach along the petrotentorial corridor without or minimal cerebellar retraction using cottonoids. Previous publications indicated retraction of the cerebellum tangential to the course of the vestibulocochlear nerve to be the preferable key maneuver as opposed to parallel to the nerve [5, 8, 23]. In our experience, the use of retractors can be avoided in most cases if the patient is adequately positioned and the prepontine cistern is opened early. Avoiding mobilization or complete detachment of the vestibulocochlear nerve from the arachnoid sheet are additional factors to be considered in order to prevent cranial nerve injury. Coagulation of cerebellar-draining veins should also be avoided to prevent venous congestion and cerebellar edema. If the view through the veins is uncertain, adequate MVD can be controlled by using an endoscope [11].

CSF fistulas and rhinoliquorrhea due to inadequate wound closure occurred in only four patients (2.3%) and resulted in prolonged hospitalization and increased risk of infection. We propose that CSF fistulas can be avoided by a tight closure of the dura mater and closure with sealing matrices, followed by close adaptation of the muscle and a tight-layered wound closure. Opened mastoid cells should be closed with autologous muscles tissue and fibrin glue. To avoid adhesions between the dura mater and the neck muscles, which may cause pain, we recommend replacing the bone flap with bone cement. Synthetic bone substitutes have also been shown to be associated with lower rates of wound-related complications [1].

### Limitations

The main limitation of this study is its retrospective nature. Furthermore, the quality of MRI images that were created on different scanners over a long period of almost 20 years differs greatly from one another and lacks a standardized protocol. The endpoint of the study represents a further limitation, because follow-up of patients was ended when pain recurrence occurred or when patients increased their pain medications. Thus, our data does not facilitate prediction of potential recurrent long-term improvements.

## Conclusions

MVD is a safe and effective treatment for refractory TGN, even in the elderly, and remains the only procedure that has the potential for cure. The morbidity profile of this procedure is low provided that the surgery is performed by a neurosurgeon trained in the surgical nuances of this special and self-rewarding operation. As patient counseling is key, TGN should be managed and treated in experienced centers that can offer all the necessary medical and surgical resources.

## Abbreviations

AICA: Anterior inferior cerebellar artery
BAEP: Brainstem auditory evoked potentials
BNI: Barrow Neurological Institute
CSF: Cerebrospinal fluid
GKN: Gamma knife radiosurgery
MVD: Microvascular decompression
MRI: Magnetic resonance imaging
NVC: Neurovascular contact
TGN: Trigeminal neuralgia
V2: Maxillary nerve
V3: Mandibular nerve

## References

[1] Alford EN, Chagoya G, Elsayed GA, Bernstock JD, Bentley JN, Romeo A, Guthrie B (2021). Risk factors for wound-related complications after microvascular decompression. Neurosurg Rev.

[2] Ariai MS, Mallory GW, Pollock BE (2014). Outcomes after microvascular decompression for patients with trigeminal neuralgia and suspected multiple sclerosis. World Neurosurg.

[3] Barker FG, Jannetta PJ, Bissonette DJ, Larkins MV, Jho HD (1996). The long-term outcome of microvascular decompression for trigeminal neuralgia. N Engl J Med.

[4] Bendtsen L, Zakrzewska JM, Abbott J, Braschinsky M, Di Stefano G, Donnet A, Eide PK, Leal PRL, Maarbjerg S, May A, Nurmikko T, Obermann M, Jensen TS, Cruccu G (2019). European Academy of Neurology guideline on trigeminal neuralgia. Eur J Neurol.

[5] Bond AE, Zada G, Gonzalez AA, Hansen C, Giannotta SL (2010). Operative strategies for minimizing hearing loss and other major complications associated with microvascular decompression for trigeminal neuralgia. World Neurosurg.

[6] Brînzeu A, Drogba L, Sindou M (2018) Reliability of MRI for predicting characteristics of neurovascular conflicts in trigeminal neuralgia: implications for surgical decision making. J Neurosurg:1–11. 10.3171/2017.8.jns171222 10.3171/2017.8.JNS171222 29624148

[7] Broggi G, Ferroli P, Franzini A, Pluderi M, La Mantia L, Milanese C (1999). Role of microvascular decompression in trigeminal neuralgia and multiple sclerosis. Lancet (London, England).

[8] Broggi G, Ferroli P, Franzini A, Servello D, Dones I (2000). Microvascular decompression for trigeminal neuralgia: comments on a series of 250 cases, including 10 patients with multiple sclerosis. J Neurol Neurosurg Psychiatry.

[9] Cruccu G, Finnerup NB, Jensen TS, Scholz J, Sindou M, Svensson P, Treede R-D, Zakrzewska JM, Nurmikko T (2016). Trigeminal neuralgia. Neurology.

[10] Devor M, Amir R, Rappaport ZH (2002). Pathophysiology of trigeminal neuralgia: the ignition hypothesis. Clin J Pain.

[11] Dubey A, Yadav N, Ratre S, Parihar VS, Yadav YR (2018). Full endoscopic vascular decompression in trigeminal neuralgia: experience of 230 patients. World Neurosurg.

[12] Dubner R, Sharav Y, Gracely RH, Price DD (1987). Idiopathic trigeminal neuralgia: sensory features and pain mechanisms. Pain.

[13] Eldridge PR, Sinha AK, Javadpour M, Littlechild P, Varma TR (2003). Microvascular decompression for trigeminal neuralgia in patients with multiple sclerosis. Stereotact Funct Neurosurg.

[14] Hai J, Li S-T, Pan Q-G (2006). Treatment of atypical trigeminal neuralgia with microvascular decompression. Neurol India.

[15] Headache Classification Committee of the International Headache Society (IHS) The International Classification of Headache Disorders, 3rd edition (2018). Cephalalgia 38:1–211. 10.1177/0333102417738202 10.1177/0333102417738202 29368949

[16] Heinskou TB, Rochat P, Maarbjerg S, Wolfram F, Brennum J, Olesen J, Bendtsen L (2019). Prognostic factors for outcome of microvascular decompression in trigeminal neuralgia: a prospective systematic study using independent assessors. Cephalalgia.

[17] Holste K, Chan AY, Rolston JD, Englot DJ (2020). Pain outcomes following microvascular decompression for drug-resistant trigeminal neuralgia: a systematic review and meta-analysis. Neurosurgery.

[18] Hussain MA, Konteas A, Sunderland G, Franceschini P, Byrne P, Osman-Farah J, Eldridge P (2018). Re-exploration of microvascular decompression in recurrent trigeminal neuralgia and intraoperative management options. World Neurosurg.

[19] Katusic S, Williams DB, Beard CM, Bergstralh EJ, Kurland LT (1991). Epidemiology and clinical features of idiopathic trigeminal neuralgia and glossopharyngeal neuralgia: similarities and differences, Rochester, Minnesota, 1945–1984. Neuroepidemiology.

[20] Koopman JS, Dieleman JP, Huygen FJ, de Mos M, Martin CG, Sturkenboom MC (2009). Incidence of facial pain in the general population. Pain.

[21] Leal PR, Froment JC, Sindou M (2010). MRI sequences for detection of neurovascular conflicts in patients with trigeminal neuralgia and predictive value for characterization of the conflict (particularly degree of vascular compression). Neurochirurgie.

[22] Lee KH, Chang JW, Park YG, Chung SS (1997). Microvascular decompression and percutaneous rhizotomy in trigeminal neuralgia. Stereotact Funct Neurosurg.

[23] McLaughlin MR, Jannetta PJ, Clyde BL, Subach BR, Comey CH, Resnick DK (1999). Microvascular decompression of cranial nerves: lessons learned after 4400 operations. J Neurosurg.

[24] Obermann M, Yoon MS, Ese D, Maschke M, Kaube H, Diener HC, Katsarava Z (2007). Impaired trigeminal nociceptive processing in patients with trigeminal neuralgia. Neurology.

[25] Olson S, Atkinson L, Weidmann M (2005). Microvascular decompression for trigeminal neuralgia: recurrences and complications. J Clin Neurosci.

[26] Paulo DL, Lopez AM, Jermakowicz WJ, Yu H, Shah H, Konrad PE, Englot DJ (2020). Microvascular decompression for trigeminal neuralgia in patients with multiple sclerosis: Predictors of treatment success. World Neurosurg.

[27] Phan K, Rao PJ, Dexter M (2016). Microvascular decompression for elderly patients with trigeminal neuralgia. J Clin Neurosci.

[28] Rogers CL, Shetter AG, Fiedler JA, Smith KA, Han PP, Speiser BL (2000). Gamma knife radiosurgery for trigeminal neuralgia: the initial experience of The Barrow Neurological Institute. Int J Radiat Oncol Biol Phys.

[29] Rughani AI, Dumont TM, Lin CT, Tranmer BI, Horgan MA (2011). Safety of microvascular decompression for trigeminal neuralgia in the elderly. Clinical article. J Neurosurg.

[30] Sandel T, Eide PK (2013) Long-term results of microvascular decompression for trigeminal neuralgia and hemifacial spasms according to preoperative symptomatology. Acta Neurochir (Wien) 155:1681–1692; discussion 1692. 10.1007/s00701-013-1816-8 10.1007/s00701-013-1816-8 23873123

[31] Sandell T, Eide PK (2010) The effect of microvascular decompression in patients with multiple sclerosis and trigeminal neuralgia. Neurosurgery 67:749–753; discussion 753–744. 10.1227/01.Neu.0000375491.81803.5d 10.1227/01.NEU.0000375491.81803.5D 20651626

[32] Sarsam Z, Garcia-Fiñana M, Nurmikko TJ, Varma TR, Eldridge P (2010). The long-term outcome of microvascular decompression for trigeminal neuralgia. Br J Neurosurg.

[33] Sekula RF, Frederickson AM, Jannetta PJ, Quigley MR, Aziz KM, Arnone GD (2011). Microvascular decompression for elderly patients with trigeminal neuralgia: a prospective study and systematic review with meta-analysis. J Neurosurg.

[34] Sindou M, Leston J, Howeidy T, Decullier E, Chapuis F (2006) Micro-vascular decompression for primary Trigeminal Neuralgia (typical or atypical). Long-term effectiveness on pain; prospective study with survival analysis in a consecutive series of 362 patients. Acta Neurochir (Wien) 148:1235–1245; discussion 1245. 10.1007/s00701-006-0809-2 10.1007/s00701-006-0809-2 16804643

[35] Tölle T, Dukes E, Sadosky A (2006). Patient burden of trigeminal neuralgia: results from a cross-sectional survey of health state impairment and treatment patterns in six European countries. Pain Pract.

[36] Tyler-Kabara EC, Kassam AB, Horowitz MH, Urgo L, Hadjipanayis C, Levy EI, Chang YF (2002). Predictors of outcome in surgically managed patients with typical and atypical trigeminal neuralgia: comparison of results following microvascular decompression. J Neurosurg.

[37] Walchenbach R, Voormolen JHC, Hermans J (1994) Microvascular decompression for trigeminal neuralgia: a critical reappraisal. 96:290-295. 10.1016/0303-8467(94)90116-3 10.1016/0303-8467(94)90116-3 7889689

[38] Yuan M, Zhou H-Y, Xiao Z-L, Wang W, Li X-L, Chen S-J, Yin X-P, Xu L-J (2016). Efficacy and safety of gabapentin vs. carbamazepine in the treatment of trigeminal neuralgia: a meta-analysis. Pain Pract.

[39] Zakrzewska JM, Thomas DG (1993) Patient’s assessment of outcome after three surgical procedures for the management of trigeminal neuralgia. Acta Neurochir (Wien) 122:225–230. 10.1007/bf01405533 10.1007/BF01405533 8372712

[40] Zakrzewska JM, Lopez BC, Kim SE, Coakham HB (2005) Patient reports of satisfaction after microvascular decompression and partial sensory rhizotomy for trigeminal neuralgia. Neurosurgery 56:1304–1311; discussion 1311–1302. 10.1227/01.neu.0000159883.35957.e0 10.1227/01.neu.0000159883.35957.e0 15918947

[41] Zakrzewska JM, Wu J, Mon-Williams M, Phillips N, Pavitt SH (2017). Evaluating the impact of trigeminal neuralgia. Pain.

[42] Zhang H, Lei D, You C, Mao BY, Wu B, Fang Y (2013). The long-term outcome predictors of pure microvascular decompression for primary trigeminal neuralgia. World Neurosurg.

